# In Situ Growth of Stable (DPPM)_2_Cu_4_I_4_@TPU Flexible Scintillator Films

**DOI:** 10.3390/s26134220

**Published:** 2026-07-03

**Authors:** Xianming Cai, Xinxin Miao, Muhammad Bilal, Ruoyu Li, Jing Li, Jun Pan

**Affiliations:** Science and Education Integration College of Energy and Carbon Neutralization, College of Materials Science and Engineering, Zhejiang University of Technology, Hangzhou 310014, China; 221123250124@zjut.edu.cn (X.C.); 221122250114@zjut.edu.cn (X.M.); mbilal@mail.ustc.edu.cn (M.B.); 211123250032@zjut.edu.cn (R.L.); lijing23@zjut.edu.cn (J.L.)

**Keywords:** copper halides, X-ray imaging, scintillators, in situ

## Abstract

Copper(I) halides are promising for X-ray scintillation owing to high luminescence and solution processability, but their poor stability limits practical use. Here we report a zero-dimensional coordinative cluster, (DPPM)_2_Cu_4_I_4_ (DPPM = bis (diphenylphosphino) methane), prepared by a simple anti-solvent crystallization that emits bright orange light with an absolute photoluminescence quantum yield of 91.11%. Spectroscopic analysis (long lifetime, large Huang–Rhys factor) indicates self-trapped-exciton dominated radiative recombination. The cluster shows outstanding thermal (stable to ≈362 °C), solvent (stable after 30 d in H_2_O, EA, EtOH, IPA) and air stability (>60 d), addressing common durability issues of copper(I) halides. Using an in situ growth method, microcrystals of (DPPM)_2_Cu_4_I_4_ were uniformly incorporated into a thermoplastic polyurethane (TPU) matrix to form flexible scintillator films. The composite exhibits a high light yield of 17,064 photons MeV^−1^ and a spatial resolution of 14 lp mm^−1^, highlighting its great potential for practical X-ray imaging applications.

## 1. Introduction

Scintillators convert high-energy radiation into ultraviolet or visible photons and are essential components in indirect X-ray detection systems for medical imaging, nondestructive testing, security screening and environmental monitoring [1,2,3,4,5,6]. Conventional commercial scintillators (e.g., CsI:TI, Lu_3_Al_5_O_12_:Ce, Bi_4_Ge_3_O_12_ and PbWO_4_) offer excellent performance but typically require high-temperature/high-pressure synthesis and costly processing, which limit scalable, low-cost manufacture for emerging applications [7,8,9,10,11]. Consequently, there is strong interest in developing novel scintillation materials that combine high performance with facile, low-temperature solution processing.

Organic-inorganic hybrid metal halides (OIMHs) have emerged as promising candidates because of their solution processability and tunable optoelectronic properties [12,13,14]. Lead-based OIMHs exhibit strong X-ray absorption and tunable emission but face commercialization barriers due to toxicity and limited environmental/thermal stability [15]. Lead-free alternatives such as manganese- and copper-based halides mitigate toxicity concerns. Manganese-based systems can provide green or red emission depending on coordination environment, but precise tuning of emission color is often challenging [16,17,18,19,20,21]. By contrast, copper(I) halides offer compositional and ligand-driven structural diversity that enables easier spectral tuning and the formation of distinct luminescent centers (e.g., Cu_2_I_4_, Cu_4_I_4_ units), making them attractive for scintillation applications [22,23,24,25,26].

Despite notable advances, many reported OIMH scintillators suffer from poor environmental, thermal or photostability, and common film fabrication methods (grinding crystalline powders and blending with polymers) often produce inhomogeneous dispersions that increase light scattering and reduce imaging resolution [27,28]. In situ growth strategies for embedding microcrystals within polymer matrices have recently shown promise for producing uniform, high-resolution scintillation films with reduced scattering. For instance, Xia et al. employed an in situ method to develop scintillation film and obtained a high spatial resolution of 14.5 lp mm^−1^ [29].

Here, we introduce a coordinative copper cluster, (DPPM)_2_Cu_4_I_4_ (DPPM = bis(diphenylphosphino)methane), synthesized via a simple anti-solvent method. The compound displays bright orange emission with a high absolute photoluminescence quantum yield (91.11%) and exceptional thermal, solvent and air stability. Using an in situ growth approach, we incorporated (DPPM)_2_Cu_4_I_4_ into a thermoplastic polyurethane (TPU) matrix to form flexible scintillator films. The resulting (DPPM)_2_Cu_4_I_4_@TPU composite demonstrates a high light yield (17,064 photons MeV^−1^), low detection limit (1.566 μGy s^−1^) and a high spatial resolution (14 lp mm^−1^), indicating its potential for practical X-ray imaging applications. The novelty of this work lies in the following: (1) the discovery of a highly stable copper(I) iodide cluster with a record PLQY of 91.11% among Cu_4_I_4_-based clusters; (2) the demonstration of excellent thermal, solvent, and air stability that addresses the common durability issues of copper(I) halides; and (3) the successful fabrication of flexible, large-area scintillator films with competitive imaging resolution through a scalable in situ growth method.

## 2. Materials and Methods

### 2.1. Chemicals and Reagents

Bis(diphenylphosphino)methane (DPPM, 98%), copper iodide (CuI,99.95%), N, N-dimethylformamide (DMF, 98%), hypophosphorous acid (H_3_PO_2_, 50wt%) and Tween 80 were purchased from Adamas Reagent Co., Ltd., Shanghai, China. Ethyl acetate (EA, AR.), ethanol (EtOH, AR.) and isopropyl alcohol (IPA,98%) were purchased from Greagent (Shanghai Titan Technology Co., Ltd., Shanghai, China). Thermoplastic polyurethane (TPU, 60-HA) was obtained from Jinhua Bojia Plastic Technology Co., Ltd., Jinhua, China. All chemicals were used as received without further purification.

### 2.2. Synthesis of (DPPM)_2_Cu_4_I_4_ Crystals

In a typical synthesis, CuI (380.8 mg, 2 mmol), DPPM (384.4 mg,1 mmol) and H_3_PO_2_ (100 μL) were dissolved in DMF (10 mL) and stirred at ambient temperature for 1 h to produce a clear precursor solution. The precursor was then added dropwise into IPA (40 mL) at room temperature to induce crystallization. The resulting microcrystalline solids were collected by vacuum filtration, washed with IPA (10 mL) and dried under vacuum at room temperature for 12 h.

### 2.3. Fabrication of (DPPM)_2_Cu_4_I_4_ @TPU Scintillation Film

The large-area scintillation film was prepared using a facile in situ growth method [30]. The TPU solution (2.0 g TPU in 10.0 mL DMF) was prepared by stirring at room temperature until complete dissolution. Amounts of 200 mg Tween 80, 380.8 mg CuI and 384.4 mg DPPM were added to this TPU/DMF precursor with stirring at ambient temperature until no visible solids remained (~1 h). The resulting (DPPM)_2_Cu_4_I_4_@TPU/DMF suspension was cast into polytetrafluoroethylene (PTFE) molds of defined area and thickness. Solvent evaporation was performed in an oven at 80 °C for 24 h under ambient pressure to yield flexible films; slower evaporation (e.g., stepwise heating or lower temperature drying) can reduce bubble formation. After drying, films were removed from the mold and stored in desiccator prior to characterization.

### 2.4. Characterization

X-ray diffraction (XRD) patterns were acquired on a diffractometer (D/max-Ultima IV) with Cu Kα radiation (λ = 1.5406 Å). Scanning electron microscopy (SEM) imaging and energy-dispersive X-ray spectroscopic (EDS) mapping were performed using a field-emission scanning electron microscope (FEI Nova45, Thermo Fisher Scientific, Hillsboro, OR, USA). UV-vis absorption spectra were recorded on a Lambda 7500 absorption spectrophotometer (PerkinElmer, Inc., Waltham, MA, USA). Photoluminescence (PL) and PL excitation spectra were collected with a fluorescence spectrophotometer (F-4600, Hitachi, Ltd., Tokyo, Japan) using an excitation wavelength of 365 nm. Absolute quantum yields were determined using a Hamamatsu Quantaurus-QY spectrometer (Hamamatsu Photonics K.K., Hamamatsu city, Japan). The XPS data were measured using a Thermo scientific K-Alpha (Thermo Fisher Scientific, Hillsboro, OR, USA). Time-resolved photoluminescence spectrum (TRPL) was measured on an Edinburgh Instruments FLS1000 (Edinburgh Instruments Ltd., Livingston, UK). FTIR spectra were recorded with a NICOLET iS50 FTIR spectrometer (Thermo Fisher Scientific, Hillsboro, OR, USA). Thermogravimetric analysis (TGA) analysis was performed under N_2_ in an alumina crucible from room temperature to 800 °C at 10 °C min^−1^ using a PerkinElmer Diamond TG/DTA6300 (PerkinElmer, Inc., Waltham, MA, USA). Radioluminescence (RL) spectra were obtained on an X-ray imaging optical system with an Amptek Mini X source (Ag target and maximum power output-4 W). The distance between the X-ray source and the sample was 5 cm, and the exposure time was 10 s. The data acquisition time for the images was about 5 s. All the measurements were conducted under the same tube voltage (50 kV), and the dose rate was modulated by varying the tube current.

## 3. Results and Discussion

**Figure 1:**
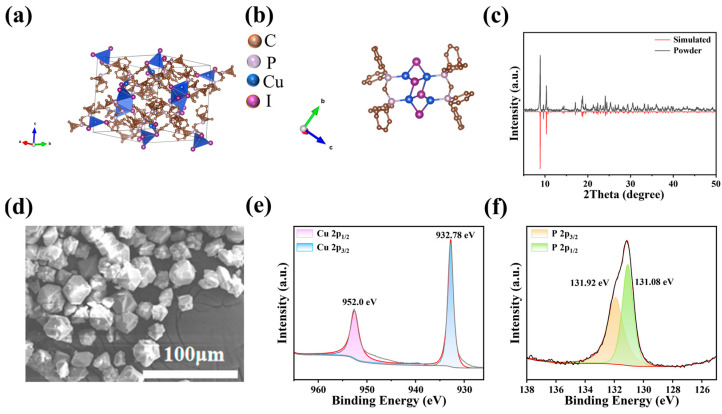
(**a**) Crystal structure of (DPPM)_2_Cu_4_I_4_. (**b**) Molecular structure of (DPPM)_2_Cu_4_I_4_. (**c**) X-ray diffraction (XRD) patterns of the simulated (DPPM)_2_Cu_4_I_4_ crystal structure, (DPPM)_2_Cu_4_I_4_ crystal powder. (**d**) Scanning electron microscopy (SEM) of the (DPPM)_2_Cu_4_I_4_@TPU film. (**e**) High resolution X-ray photoelectron spectrum of the Cu 2p core-level region. (**f**) High-resolution X-ray photoelectron spectrum of the P 2p core-level region.

(DPPM)_2_Cu_4_I_4_ microcrystals were obtained via anti-solvent assisted crystallization method [31], as illustrated in Figure S1 (Supporting Information). Briefly, bis(diphenylphosphino)methane (DPPM) and copper(I) iodide (CuI) were dissolved in N, N-dimethylformamide (DMF) with a small amount of hypophosphorous acid (H_3_PO_2_), followed by stirring at room temperature. The precursor solution was slowly dropped into the antisolvent isopropyl alcohol (IPA) to induce crystallization. Single-crystal data (CCDC 1132845) indicate an orthorhombic unit cell (space group Pbca) with a = 17.13 Å, b = 18.31 Å, c = 16.51 Å (α = β = γ = 90°) [32]. As shown in Figure 1a,b, the structure consists of discrete [Cu_4_I_4_] tetranuclear units embedded in a DPPM organic framework, and Cu atoms coordinate both I and P atoms to form a robust coordination network. This 0D molecular arrangement and Cu–P coordination likely contribute to the enhanced stability of the compound. Powder XRD of the as-synthesized material matches the simulated pattern from the single-crystal structure (Figure 1c), confirming phase purity. Additionally, the morphology of (DPPM)_2_Cu_4_I_4_ (Figure 1d) is illustrated by the scanning electron microscopy (SEM), which exhibits a polyhedral microstructure with an average diameter of 11.2 μm in length (Figure S2). Energy dispersive spectrometry (EDS) elemental maps confirm uniform distribution of P, Cu and I (Figure S3).

The surface binding states of (DPPM)_2_Cu_4_I_4_ powders were examined by X-ray photoelectron spectroscopy (XPS), with the C 1s peak at 284.8 eV used as the internal reference for calibration. The survey spectrum (Figure S4a) clearly shows characteristic peaks corresponding to Cu, I and P. Further analysis of the copper valence state was performed using high-resolution XPS (HRXPS). The Cu 2p spectrum exhibits only the Cu 2p_3/2_ peak at 932.78 eV and the Cu 2p_1/2_ peak at 952.0 eV without Cu^2+^ satellite features (Figure 1e), confirming the Cu(I) oxidation state [33]. I 3d and P 2p core levels appear at expected binding energies (Figure S4 and Figure 1f), while FTIR shows vibrational features consistent with DPPM coordination (Figure S5). Together these data indicate intact organic ligand coordination and absence of significant surface oxidation.

**Figure 2:**
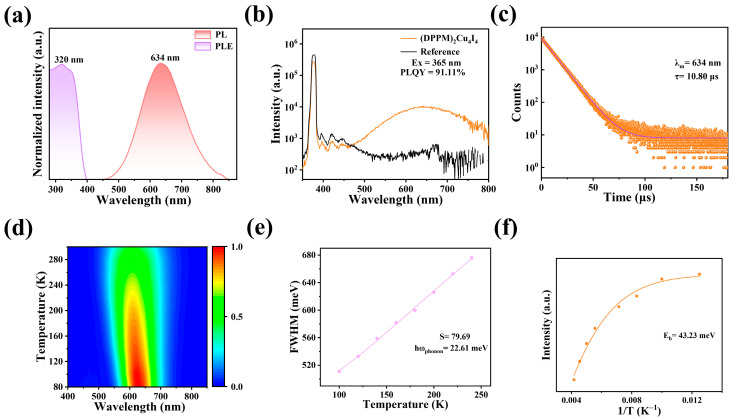
(**a**) Photoluminescence (PL) emission and photoluminescence excitation (PLE) spectra of (DPPM)_2_Cu_4_I_4_. (**b**) Photoluminescence quantum yield (PLQY) spectra of (DPPM)_2_Cu_4_I_4_ under 365 nm excitation. (**c**) Room-temperature PL decay curve (λem = 634 nm), the purple solid line represents the fitting result. (**d**) 2D view of temperature-dependent PL spectra. (**e**) PL FWHM vs. temperature with Huang–Rhys fit. (**f**) PL intensity vs. 1/T with Arrhenius fit for exciton binding energy.

To investigate the luminescence characteristics of (DPPM)_2_Cu_4_I_4_, photoluminescence (PL) and photoluminescence excitation (PLE) spectra were acquired. As depicted in Figure 2a, the PLE spectrum shows a pronounced excitation peak at 320 nm. Upon excitation at 320 nm, (DPPM)_2_Cu_4_I_4_ displays a broad emission band centered at 634 nm, with a full width at half maximum (FWHM) of 163 nm and a Stokes shift of 314 nm. This substantial Stokes shift effectively suppresses self-absorption, rendering (DPPM)_2_Cu_4_I_4_ highly promising for scintillation imaging applications. Notably, the PL spectrum exhibits a single emission band with a high absolute photoluminescence quantum yield (PLQY) of 91.11% under 365 nm excitation (Figure 2b). The UV–vis absorption spectrum of (DPPM)_2_Cu_4_I_4_ powder reveals strong absorption below 356 nm, and a Tauc plot yields a bandgap value of 3.23 eV (Figure S6). Furthermore, the time-resolved PL decay curve monitored at 634 nm follows a mono-exponential decay with a fitted lifetime of 10.8 μs at room temperature, consistent with radiative recombination of long-lived self-trapped excitons (STEs) typical of 0D Cu(I) halides (Figure 2c).

To probe the emission mechanism, PL spectra of (DPPM)_2_Cu_4_I_4_ were measured under excitation from 260 to 360 nm (Figure S7). The emission peak position was invariant with excitation wavelength, indicating a common excited state. Temperature-dependent PL spectra of (DPPM)_2_Cu_4_I_4_ (80–380 K, Figure 2d) shows decreasing intensity and broadening FWHM with increasing temperature, consistent with enhanced nonradiative processes and stronger phonon coupling [34]. As shown in Figure S8, a blue shift of the emission peak was observed with increasing temperature, which may be attributed to enhanced electron–phonon interactions that reduce the bandgap. Furthermore, the absence of peak splitting at low temperatures indicates a single radiative pathway [35,36]. The Huang–Rhys factor (*S*), quantifying electron-phonon coupling, was obtained from the FWHM versus temperature data (Figure 2e) using the following equation [37]:

(1) $$FWHM=2.36\sqrt{S}\;{h\omega }_{phonon}\sqrt{\coth\frac{{h\omega }_{phonon}}{2K_{B}T}}$$

where *hω_phonon_* represents phonon energy [38]. Fitting the curve yielded an “*S*” factor of 79.69 for (DPPM)_2_Cu_4_I_4_, indicating strong electron–phonon coupling [39].

The exciton binding (*E_b_*) energy was extracted from the temperature-dependent PL intensity using an Arrhenius equation [40,41].

(2) $$I\left(T\right)=\frac{I_{0}}{1+A\exp\left(-\frac{E_{b}}{K_{B}T}\right)}$$

where *I(T)* represents the PL emission intensity at different temperatures, I_0_ is the PL emission intensity at 0 K, A is the proportionality constant, and *K_B_* is the Boltzmann constant. Figure 2f shows the relationship between the PL intensity and the reciprocal of the temperature. The calculated *E_b_* was 43.23 meV, which exceeds both the exciton binding energy of traditional three-dimensional perovskites [42] and the thermal energy under ambient conditions (26 meV). Large values of electron–phonon coupling strength (*S*) and exciton binding energy (*E_b_*) favor efficient radiative recombination of STEs [43,44].

Material stability is an important index for practical applications, while generally poor stabilities have been reported for most OIMHs. To evaluate the thermal stability of (DPPM)_2_Cu_4_I_4_, thermogravimetric analysis was performed, as presented in Figure S9. The results show that the (DPPM)_2_Cu_4_I_4_ powder remains stable with no mass loss below 362 °C, demonstrating good thermal stability. Upon further heating, decomposition begins above 362 °C. At 456 °C, the powder exhibits a mass loss of 50.8% of its initial weight, which corresponds to the degradation of DPPM and aligns closely with the theoretical value of 50%. In the second stage, a mass loss of 31.8% is observed, in good agreement with the theoretical iodine content of 33.2%, and can therefore be assigned to the loss of iodine. These findings further validate the expected compositional ratio of DPPM to CuI (1:2) in (DPPM)_2_Cu_4_I_4_.

To verify the solvent stability of as-synthesized (DPPM)_2_Cu_4_I_4_, we soaked the (DPPM)_2_Cu_4_I_4_ powders separately in H_2_O, EA, EtOH and IPA. After being immersed for 30 days, all test samples exhibit similar XRD patterns identical to the fresh microcrystals (Figure S10a). These samples retained PL intensities of ~91.2% (H_2_O), 97.6% (EA), 83.5% (EtOH) and 88.9% (IPA) (Figure S10b). PLQY under continuous 365 nm irradiation is stable (Figure S10d), indicating good photostability. Additionally, powder stored in air for 60 days shows negligible XRD change (Figure S11). The combined thermal, solvent and air stability likely derives from the strong Cu–P coordination and the encapsulating DPPM environment.

**Figure 3:**
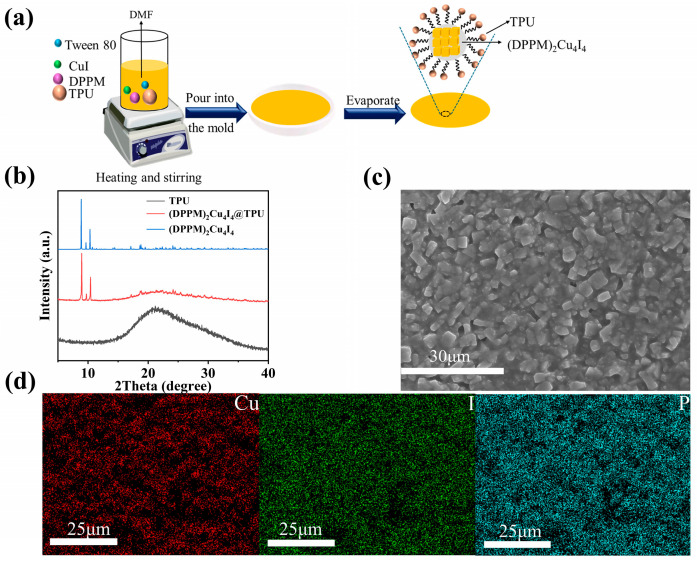
(**a**) Schematic diagram of (DPPM)_2_Cu_4_I_4_@TPU film preparation process. (**b**) X-ray diffraction (XRD) patterns of (DPPM)_2_Cu_4_I_4_ crystals, (DPPM)_2_Cu_4_I_4_@TPU film, and TPU. (**c**) Scanning electron microscopy (SEM) of the (DPPM)_2_Cu_4_I_4_@TPU film. (**d**) Energy dispersive X-ray spectroscopy (EDS) mapping of (DPPM)_2_Cu_4_I_4_@TPU film.

The (DPPM)_2_Cu_4_I_4_@TPU film was fabricated using a straightforward in situ method, as illustrated in Figure 3a. Under visible light, the (DPPM)_2_Cu_4_I_4_ crystals dispersed in the TPU matrix retain their intrinsic white color, whereas the films display bright orange emission upon UV excitation (Figure S12a,b). The PLQYs of (DPPM)_2_Cu_4_I_4_@TPU films from three independent batches were measured, yielding an average value of 53.14% (Figure S13). Owing to the superior strength and toughness of TPU compared to other plastics, the flexible film withstood mechanical deformation without physical damage (Figure S12c,d). Tensile tests were further conducted to evaluate the mechanical properties of the (DPPM)_2_Cu_4_I_4_@TPU scintillation film (Figure S14a). The composite film remained intact after folding and could even be stretched up to 400% without cracking. Moreover, the PL intensity was preserved under stretching, and no shift in the PL peak position was observed with increasing elongation (Figure S14b), indicating that the material meets the requirements for use in flexible devices.

To confirm the crystalline composition within the TPU matrix, XRD analysis was performed on the as-prepared (DPPM)_2_Cu_4_I_4_@TPU film, along with pristine (DPPM)_2_Cu_4_I_4_ powder and TPU. As shown in Figure 3b, the XRD patterns of both the microcrystalline powder and pure TPU are well-matched with those reported in the literature [32]. The diffraction peaks of the (DPPM)_2_Cu_4_I_4_@TPU scintillation film correspond to those of the (DPPM)_2_Cu_4_I_4_ spectrum, albeit with differences in intensity and the attenuation of some peaks. The morphology and elemental distribution of the composite film were further characterized by SEM and EDS. As shown in Figure 3c, the SEM image reveals uniform growth of microcrystals within the TPU matrix. Corresponding EDS mapping (Figure 3d) confirms the homogeneous dispersion of Cu, I and P throughout the film. This uniform elemental distribution was further verified across multiple sampled regions, as demonstrated in Figure S15, confirming the good structural and compositional homogeneity of the as-prepared scintillation film.

**Figure 4:**
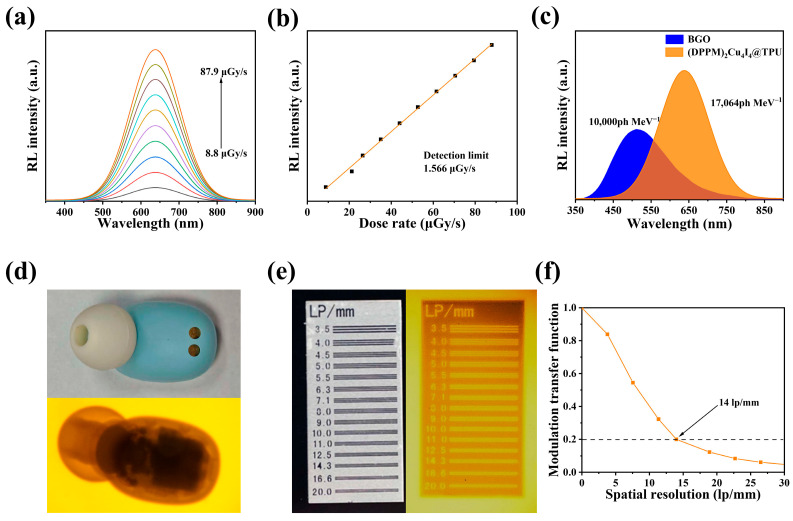
(**a**) Dosage-dependent radioluminescence (RL) spectra of in situ grown (DPPM)_2_Cu_4_I_4_@TPU film. (**b**) Detection limit (DL) of the in situ grown (DPPM)_2_Cu_4_I_4_@TPU film. (**c**) RL spectrum of (DPPM)_2_Cu_4_I_4_@TPU film compared to the traditional scintillator Bi_4_Ge_3_O_12_ (BGO). (**d**) Physical image and X-ray image of Bluetooth headset. (**e**) Physical image and X-ray image of a Pb-based X-ray resolution scale. (**f**) Modulation transfer function curve of in situ grown (DPPM)_2_Cu_4_I_4_@TPU scintillator film.

Radioluminescence (RL) spectra of the (DPPM)_2_Cu_4_I_4_@TPU film measured at various X-ray dose rates (Figure 4a) show an orange emission whose peak position and FWHM match the PL spectra under UV excitation, indicating the same radiative recombination pathway. The strong RL spectra also confirm its suitability as an X-ray imaging scintillator. As the X-ray dose rate was increased from 8.8 µGy s^−1^ to 87.9 µGy s^−1^, the RL intensity rose proportionally, demonstrating a linear response (Figure 4b). Based on the slope of the linear fit, the detection limit of the film was calculated to be 1.566 μGy s^−1^. The light yield was evaluated by comparing the RL intensity of the (DPPM)_2_Cu_4_I_4_@TPU film with that of a commercial BGO scintillator, which has a known light yield of 10,000 photons MeV^−1^ (Figure 4c). The composite film achieved a light yield of 17,064 photons MeV^−1^, which is about 1.7 times that of the BGO standard. For a more practical comparison, the light yield of our film is still lower than that of the commercial CsI:Tl scintillator (approximately 54,000 photons MeV^−1^) [45], which is widely used in medical X-ray imaging. Nevertheless, our (DPPM)_2_Cu_4_I_4_@TPU film offers advantages in terms of solution processability, flexibility and low-cost fabrication, making it a promising candidate for applications where mechanical flexibility and ease of manufacturing are prioritized. RL measurements for different batches of (DPPM)_2_Cu_4_I_4_@TPU films are presented in Figure S16a, with the corresponding light yields shown in Figure S16b. The average light yield across all batches is 17,211 photons MeV^−1^, demonstrating reliable scintillation performance and good reproducibility of the (DPPM)_2_Cu_4_I_4_@TPU film for X-ray imaging.

Radiation stability of the (DPPM)_2_Cu_4_I_4_@TPU scintillation film was evaluated under continuous X-ray irradiation. As shown in Figure S17, the RL intensity remained stable during 10 min of continuous exposure at 1.221 mGy s^−1^, indicating strong radiation resistance. The X-ray imaging configuration, incorporating a Bluetooth headset and a Pb-based resolution scale, is illustrated in Figure S18. The resulting X-ray images (Figure 4d,e and Figure S19) were clear and well-resolved. Spatial resolution, obtained from a standard Pb-based resolution scale using the modulation transfer function (MTF) method [46], was measured to be 14 lp mm^−1^ (Figure 4f). Two additional batches yielded resolutions of 12.7 and 13.8 lp mm^−1^, respectively (Figure S20), further confirming reproducible fabrication and imaging performance. This value corresponds well with the observed image quality, demonstrating the practical imaging capability of the (DPPM)_2_Cu_4_I_4_@TPU film. Compared with recently reported scintillators listed in Table S1, the (DPPM)_2_Cu_4_I_4_@TPU film exhibits comparable performance, confirming its potential as an advanced material for next-generation high-performance X-ray scintillators.

## 4. Conclusions

In summary, we have developed a cuprous crystal structured on discrete tetranuclear units, formulated as (DPPM)_2_Cu_4_I_4_. This crystal exhibits a high photoluminescence quantum yield of 91.11%, large Stokes shifts and outstanding stability. Its luminescence originates from self-trapped exciton emission. Using an in situ method, we fabricated a scintillator film by incorporating the crystal into a TPU matrix. The resulting film demonstrates excellent linear response to X-rays, a steady-state light yield of 17,064 photons MeV^−1^ and a detection limit as low as 1.566 μGy s^−1^. Furthermore, the scintillator film achieves a remarkable imaging resolution of 14 lp mm^−1^, highlighting its strong potential for applications in X-ray medical radiography and nondestructive testing.

## Data Availability

The data are not publicly available due to the relevant project regulations.

## Supplementary Materials

The following supporting information can be downloaded at https://www.mdpi.com/article/10.3390/s26134220/s1: Figure S1: Schematic diagram of a synthesis of (DPPM)_2_Cu_4_I_4_; Figure S2: Size distribution diagram of (DPPM)_2_Cu_4_I_4_; Figure S3: (a) SEM image of (DPPM)_2_Cu_4_I_4_ powders. EDS mapping of (DPPM)_2_Cu_4_I_4_ powders: (b) Cu, (c) I, (d) P; Figure S4: (a) XPS spectra of (DPPM)_2_Cu_4_I_4_, (b) HRXPS spectrum of I; Figure S5: FTIR spectra of (DPPM)_2_Cu_4_I_4_ and DPPM powders; Figure S6: Absorption spectrum of (DPPM)_2_Cu_4_I_4_ powder (inset: calculated band gap derived from the absorption spectrum); Figure S7: PL intensity of (DPPM)_2_Cu_4_I_4_ under different excitation wavelengths from 260 nm to 360 nm; Figure S8: Temperature-dependent PL spectra of (DPPM)_2_Cu_4_I_4_ in the range of 80–380 K; Figure S9: Thermogravimetric curve of the (DPPM)_2_Cu_4_I_4_ powders; Figure S10: (a) XRD patterns of fresh (DPPM)_2_Cu_4_I_4_ powders and after soaked in different solvents for 30 days, (b) PL spectra of fresh (DPPM)_2_Cu_4_I_4_ powders and after soaked in different solvents for 30 days, (c) XRD patterns of fresh (DPPM)_2_Cu_4_I_4_ powders and after UV irradiation for 24 h, (d) The relative PLQY of the (DPPM)_2_Cu_4_I_4_ powders as a function of irradiation time; Figure S11: XRD patterns of (DPPM)_2_Cu_4_I_4_ before and after stored in air for 2 months; Figure S12: Photographs of the (DPPM)_2_Cu_4_I_4_@TPU scintillation films (a) under visible light and (b) under 365 nm irradiation, photographs of the (DPPM)_2_Cu_4_I_4_@TPU scintillation film with mechanical deformation (c) under visible light and (d) under UV excitation; Figure S13: PLQY measurements of different batches of (DPPM)_2_Cu_4_I_4_@TPU scintillation films.: (a) sample 1, (b) sample 2, (c) sample 3; Figure S14: (a) photographs of (DPPM)_2_Cu_4_I_4_@TPU scintillation film at different elongation under UV 365 nm, (b) normalized PL spectra of (DPPM)_2_Cu_4_I_4_@TPU scintillation film at different elongation; Figure S15: Element distribution maps of Cu, I and P in multiple regions of (DPPM)_2_Cu_4_I_4_@TPU scintillation film: (a) area 1, (b) area 2, (c) area 3; Figure S16: (a) RL curves of different batches of (DPPM)_2_Cu_4_I_4_@TPU scintillation film, (b) the light yields of different batches of (DPPM)_2_Cu_4_I_4_@TPU scintillation films calculated from the RL curves; Figure S17: Changes in RL intensity of (DPPM)_2_Cu_4_I_4_@TPU scintillator film under 10 min continuous X-ray irradiation at a dose rate of 1.221 mGy s^−1^; Figure S18: Schematic diagram of our self-built X-ray imaging system; Table S1: Performance and materials characteristics of scintillators; Figure S19: X-ray images of a Pb-based resolution scale, captured using two batches of (DPPM)_2_Cu_4_I_4_@TPU scintillation films; Figure S20: Modulation transfer functions of two batches of (DPPM)_2_Cu_4_I_4_@TPU scintillation films: (a) sample 1, (b) sample 2. References [5,13,18,29,41,47,48,49,50,51] are cited in the supplementary materials.
