# Supplementary material for: In Situ Growth of Stable (DPPM)2Cu4I4@TPU Flexible Scintillator Films
Source: Sensors (Basel). 2026 Jul 3;26(13):4220. doi: 10.3390/s26134220 (PMC13363807; doi:10.3390/s26134220)
Supplement: Supplementary file 1 [file sensors-26-04220-s001.zip › sensors-4332573-supplementary.pdf]

Supporting Information

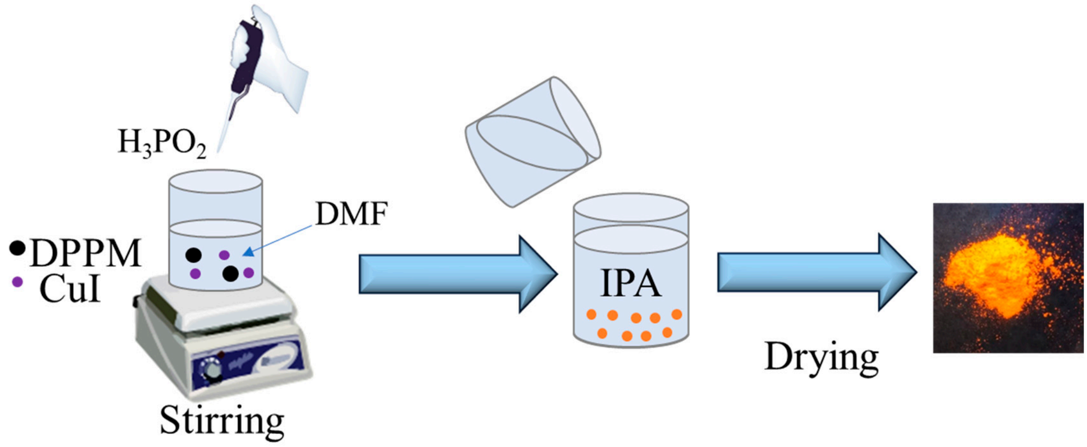

Figure S1. Schematic diagram of a synthesis of $(DPPM)_2Cu_4I_4$.

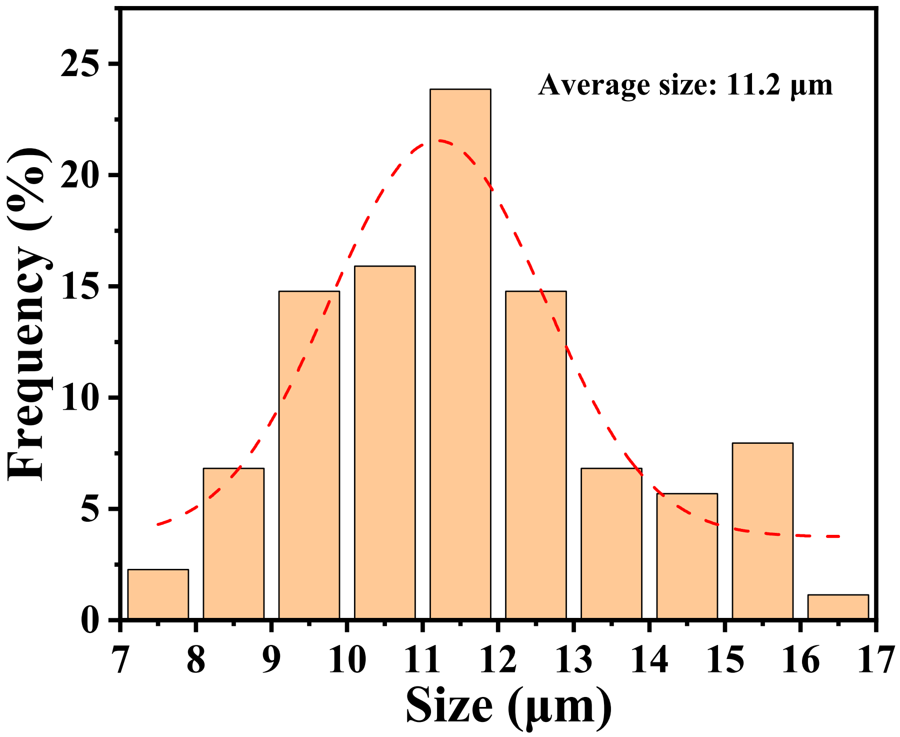

Figure S2. Size distribution diagram of $(DPPM)_2Cu_4I_4$.

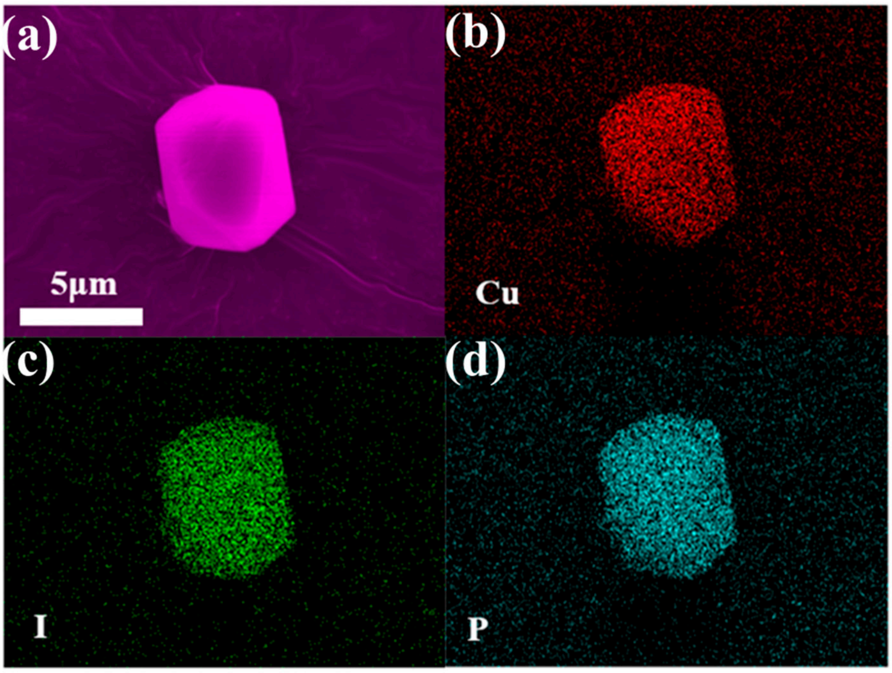

Figure S3. (a) SEM image of $(DPPM)_2Cu_4I_4$ powders. EDS mapping of $(DPPM)_2Cu_4I_4$ powders :(b) Cu, (c) I, (d) P.

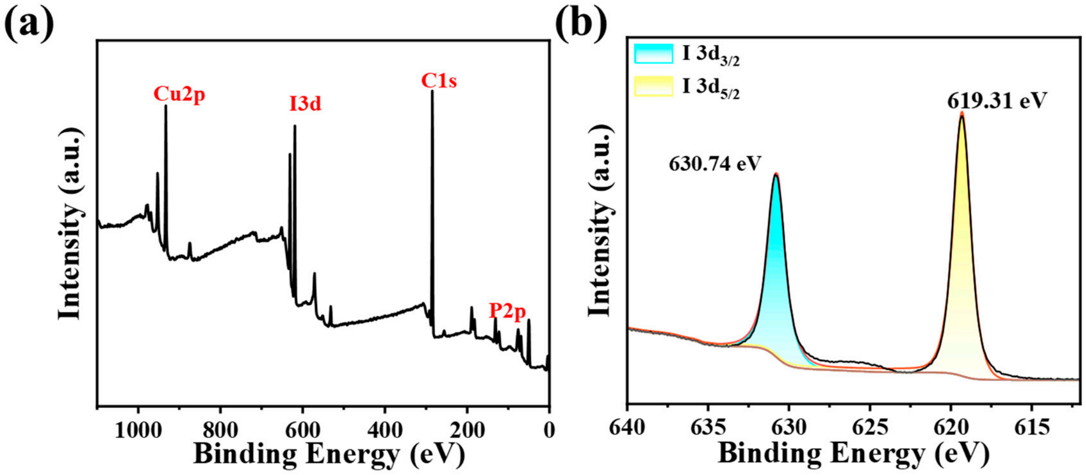

Figure S4. (a) XPS spectra of $(DPPM)_2Cu_4I_4$; (b) HRXPS spectrum of I.

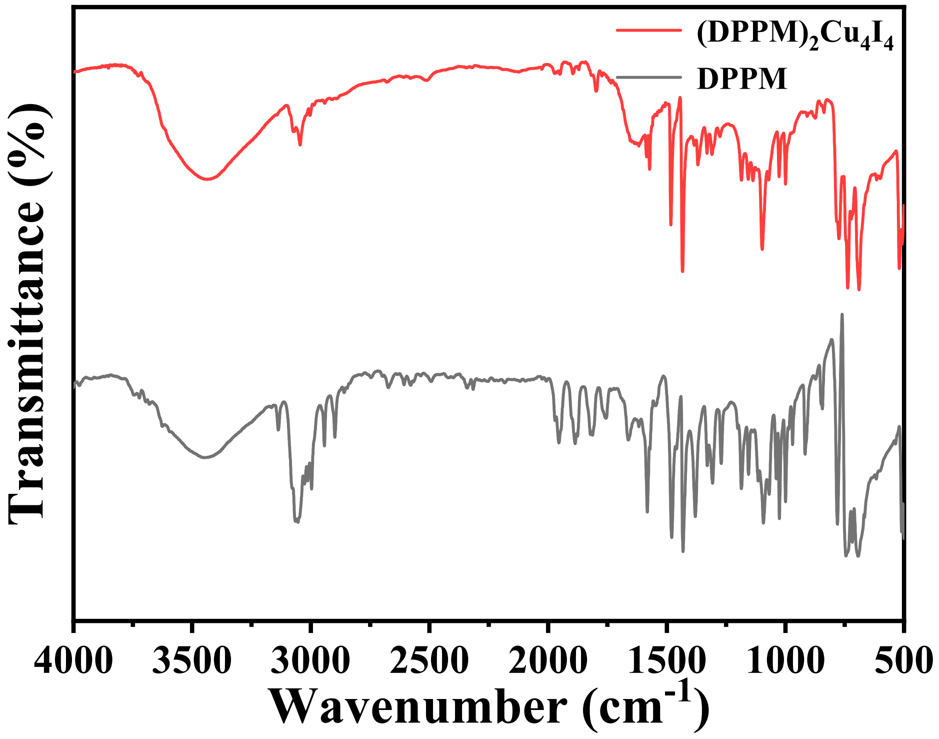

Figure S5. FTIR spectra of $(DPPM)_2Cu_4I_4$ and DPPM powders.

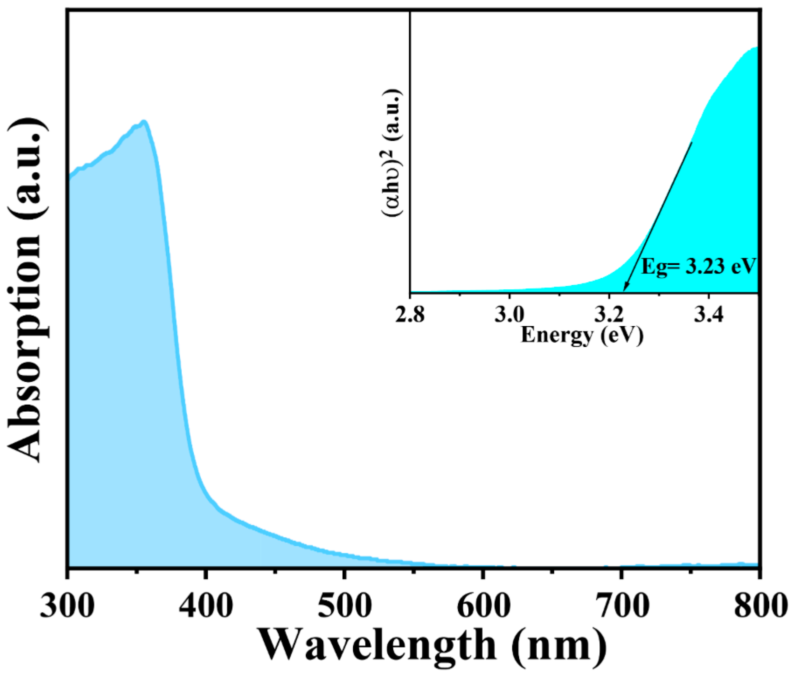

Figure S6. Absorption spectrum of $(DPPM)_2Cu_4I_4$ powder (inset: calculated band gap derived from the absorption spectrum).

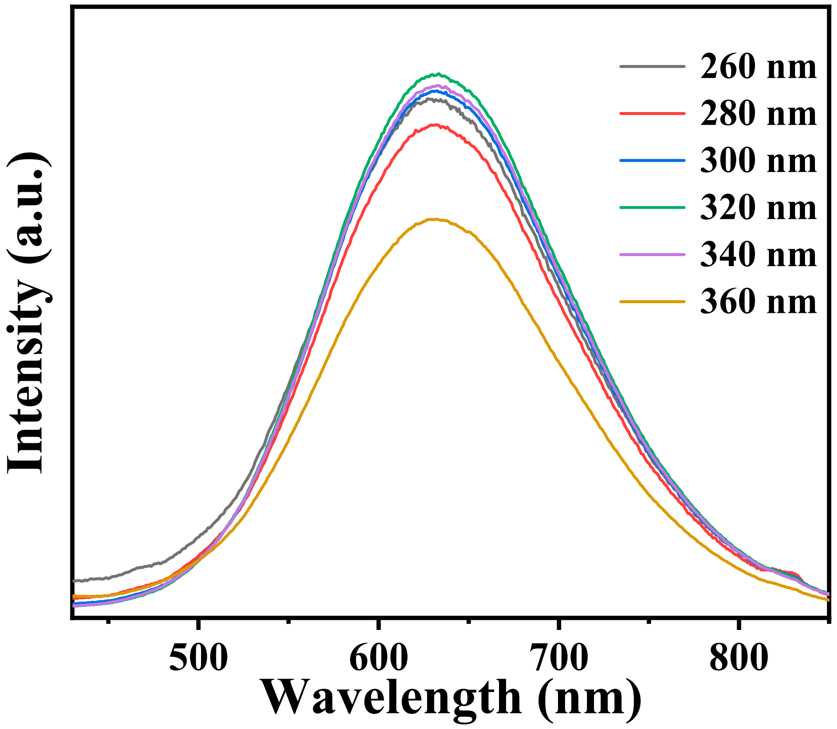

Figure S7. PL intensity of $(DPPM)_2Cu_4I_4$ under different excitation wavelengths from 260 nm to 360 nm.

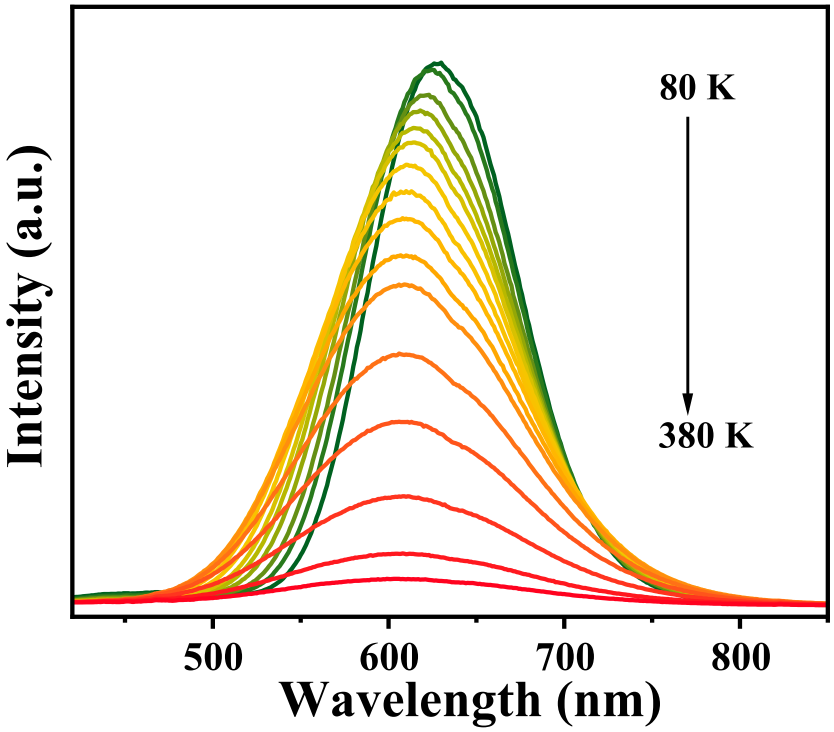

Figure S8. Temperature-dependent PL spectra of $(DPPM)_2Cu_4I_4$ in the range of 80–380 K.

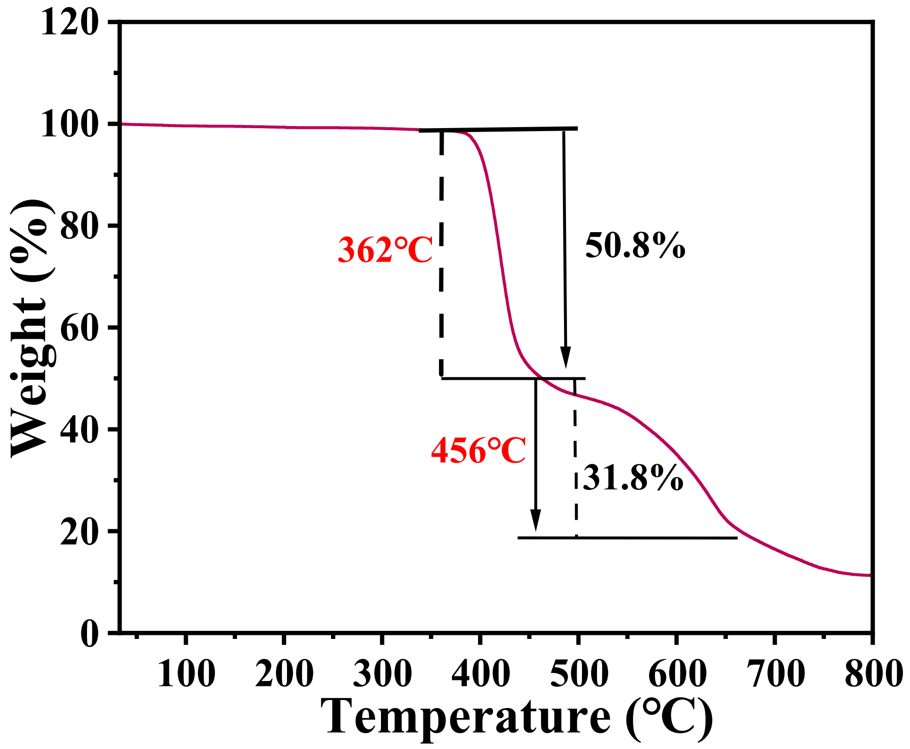

Figure S9. Thermogravimetric curve of the $(DPPM)_2Cu_4I_4$ powders.

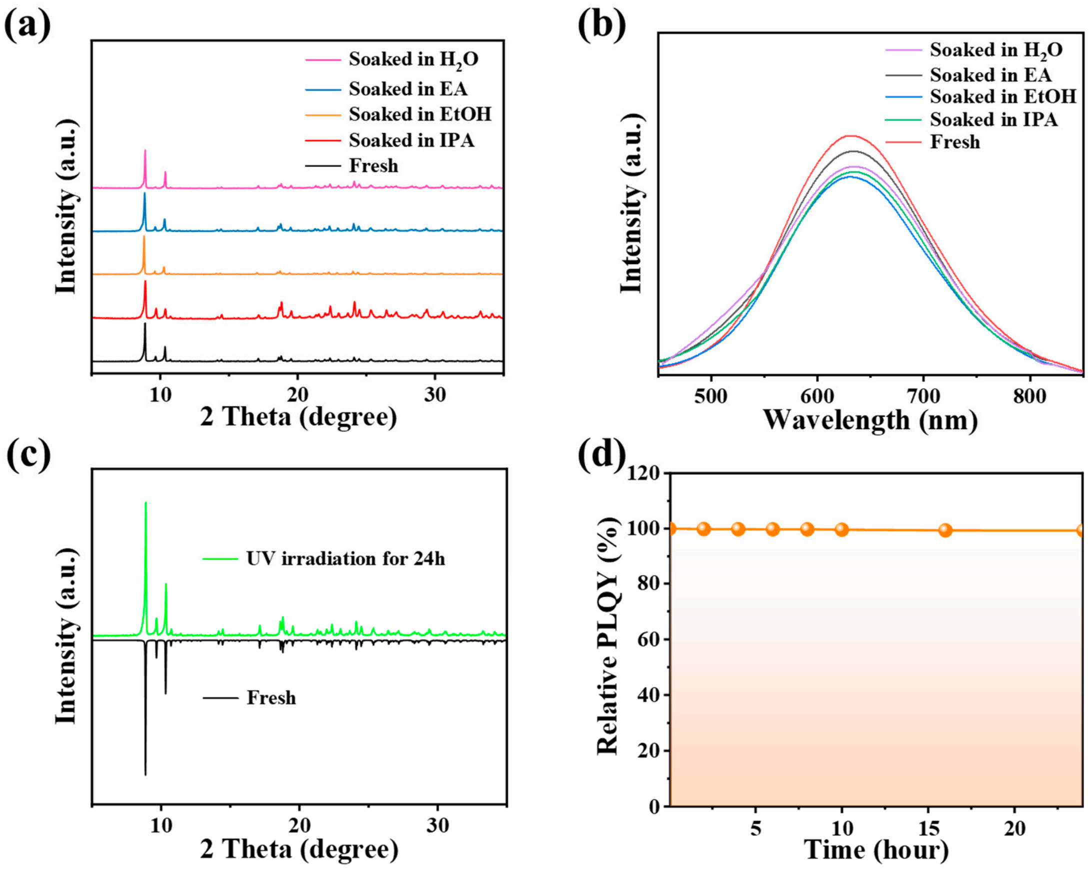

Figure S10. (a) XRD patterns of fresh $(DPPM)_2Cu_4I_4$ powders and after soaked in different solvents for 30 days; (b) PL spectra of fresh $(DPPM)_2Cu_4I_4$ powders and after soaked in different solvents for 30 days; (c) XRD patterns of fresh $(DPPM)_2Cu_4I_4$ powders and after UV irradiation for 24 hours; (d) The relative PLQY of the $(DPPM)_2Cu_4I_4$ powders as a function of irradiation time.

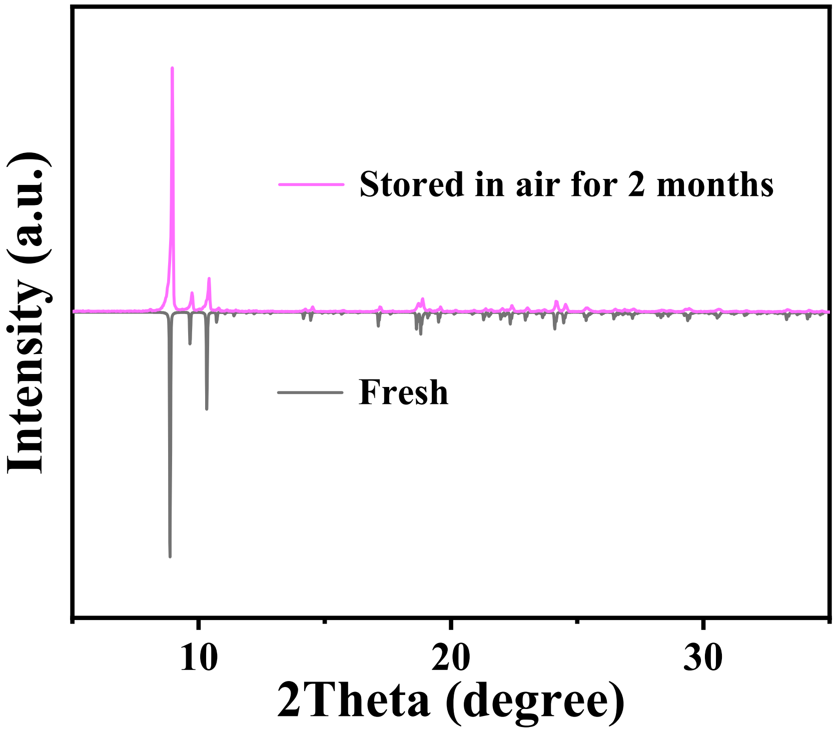

Figure S11. XRD patterns of $(DPPM)_2Cu_4I_4$ before and after stored in air for 2 months.

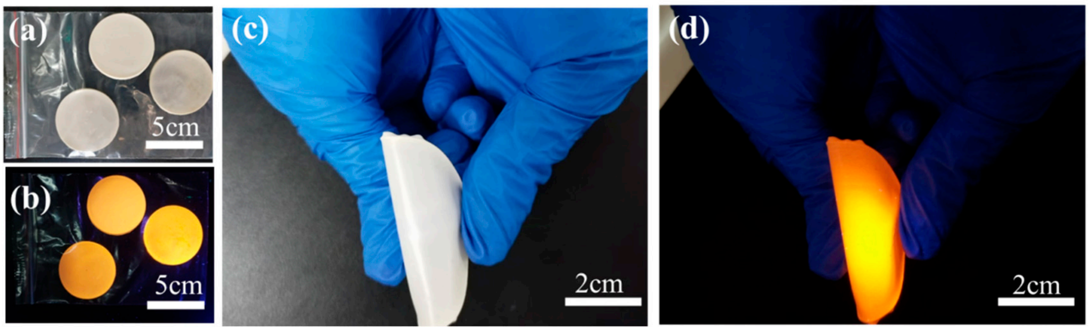

Figure S12. Photographs of the $(DPPM)_2Cu_4I_4$@TPU scintillation films: (a) under visible light and (b) under 365 nm irradiation; Photographs of the $(DPPM)_2Cu_4I_4$@TPU scintillation film with mechanical deformation: (c) under visible light and (d) under UV excitation.

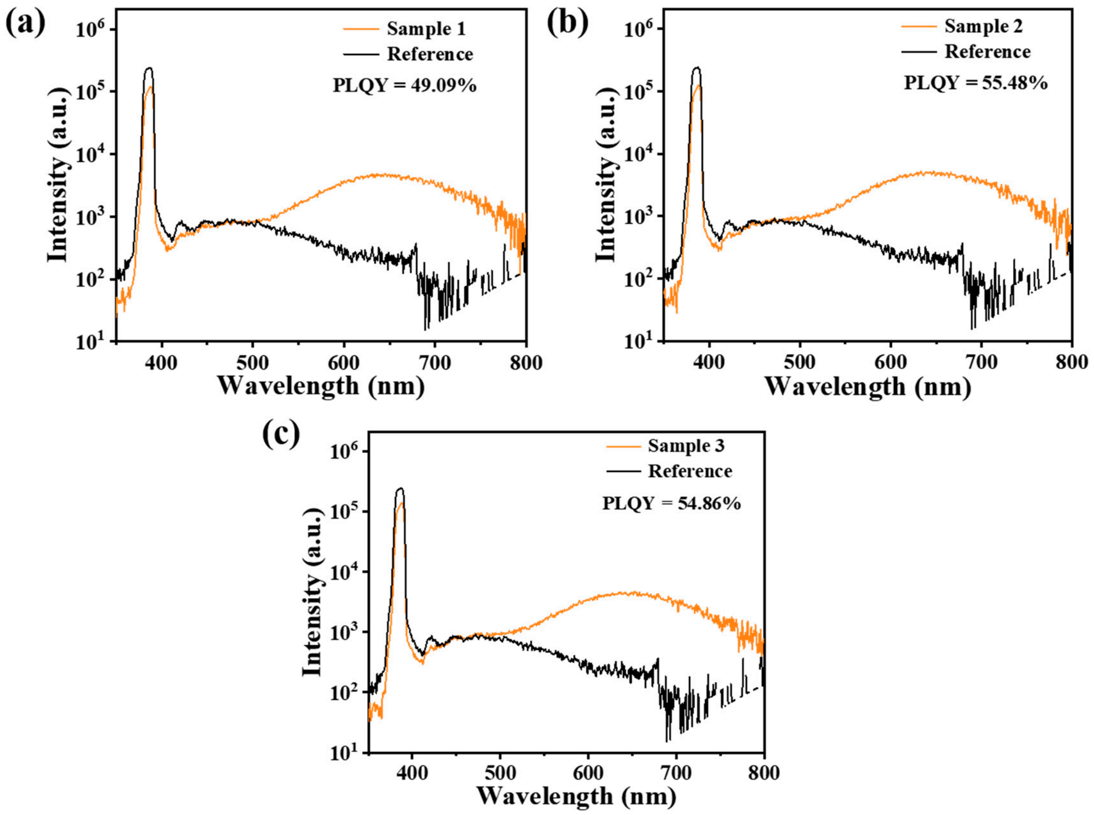

Figure S13. PLQY measurements of different batches of $(DPPM)_2Cu_4I_4$@TPU scintillation films.: (a) sample 1, (b) sample 2, (c) sample 3.

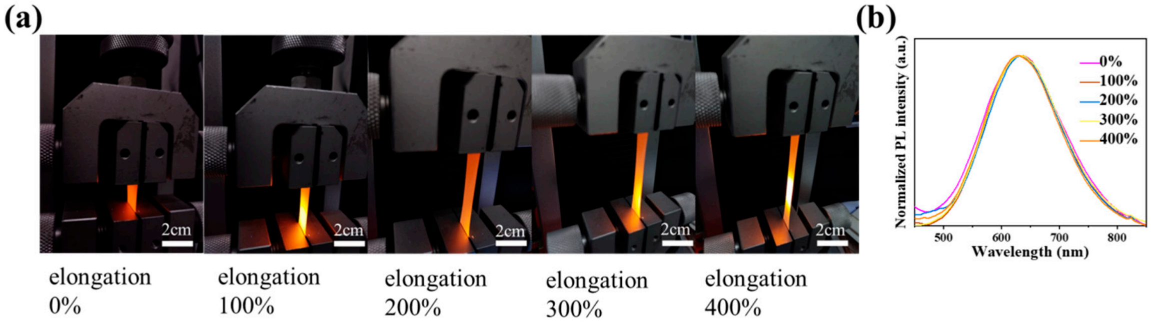

Figure S14. (a) Photographs of $(DPPM)_2Cu_4I_4$@TPU scintillation film at different elongation under UV 365 nm; (b) Normalized PL spectra of $(DPPM)_2Cu_4I_4$@TPU scintillation film at different elongation.

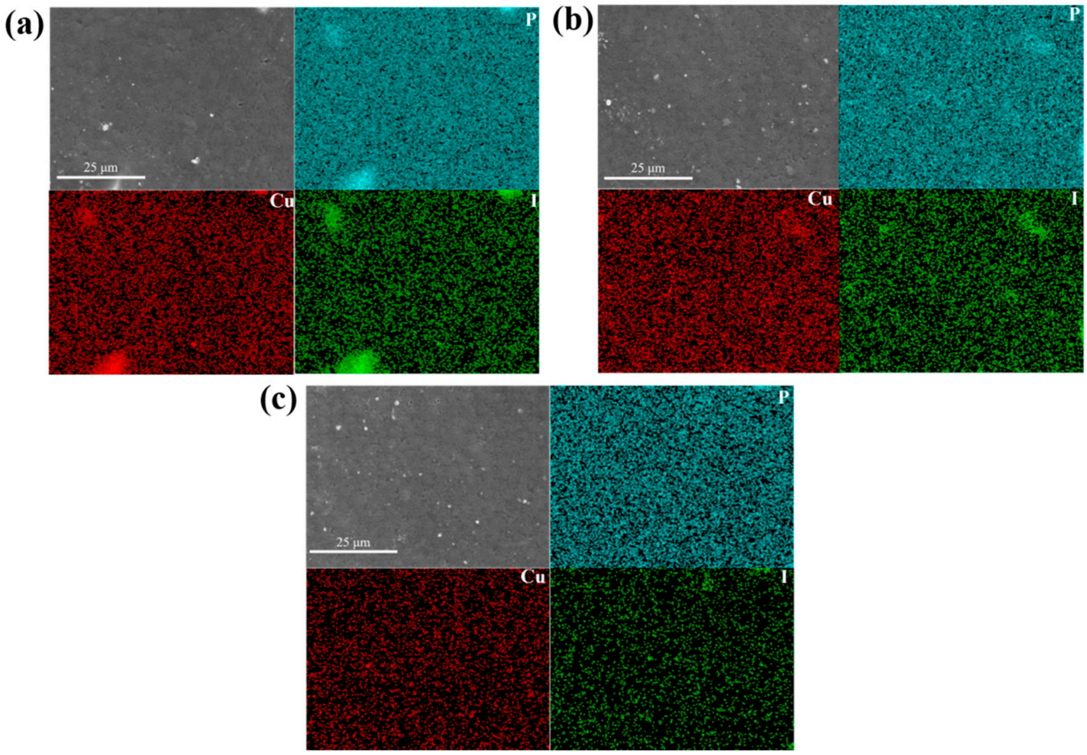

Figure S15. Element distribution maps of Cu, I, and P in multiple regions of $(DPPM)_2Cu_4I_4$@TPU scintillation film: (a) area 1, (b) area 2, (c) area 3.

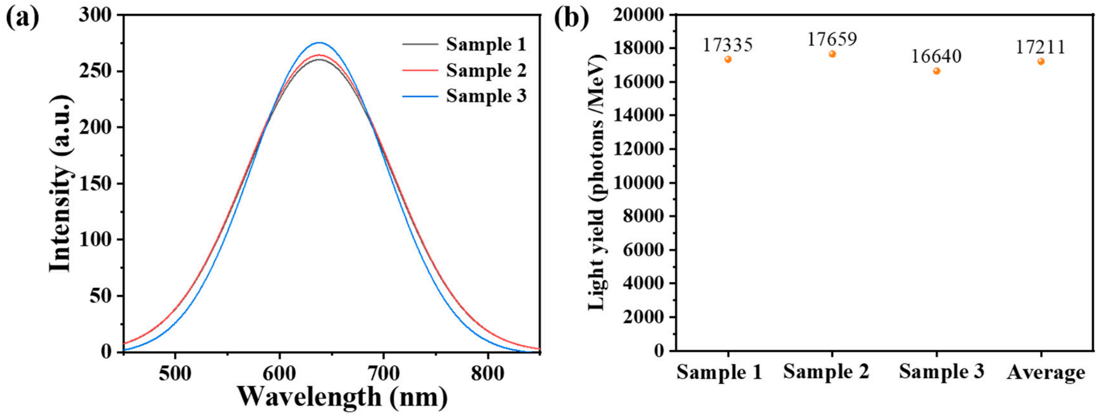

Figure S16. (a) RL curves of different batches of $(DPPM)_2Cu_4I_4$@TPU scintillation film; (b) Light yields of different batches of $(DPPM)_2Cu_4I_4$@TPU scintillation films (calculated from the RL curves).

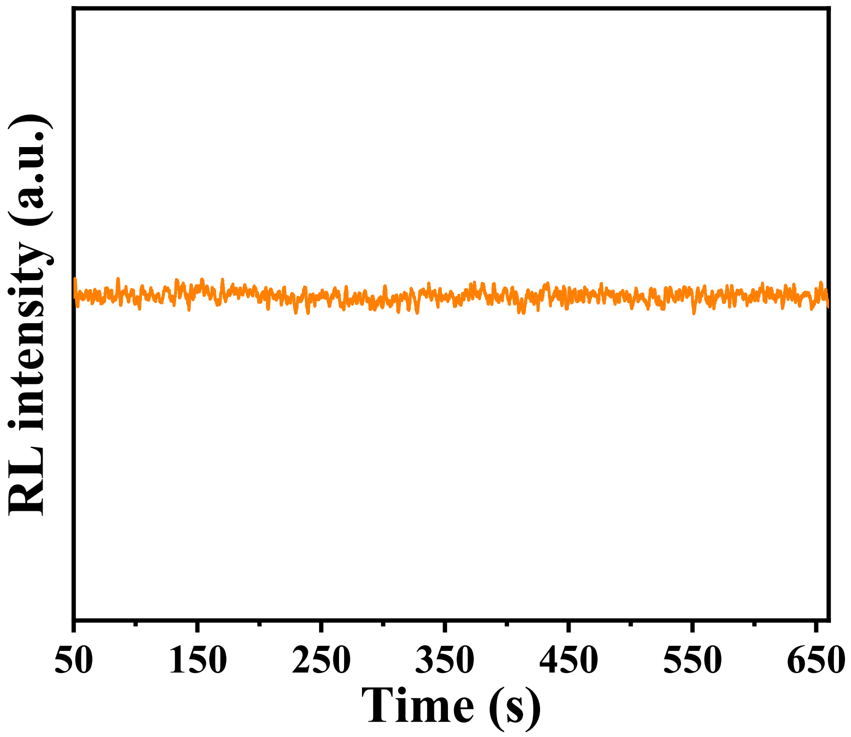

Figure S17. Changes in RL intensity of $(DPPM)_2Cu_4I_4$@TPU scintillator film under 10 min continuous X-ray irradiation at a dose rate of 1.221 mGy s^{-1}.

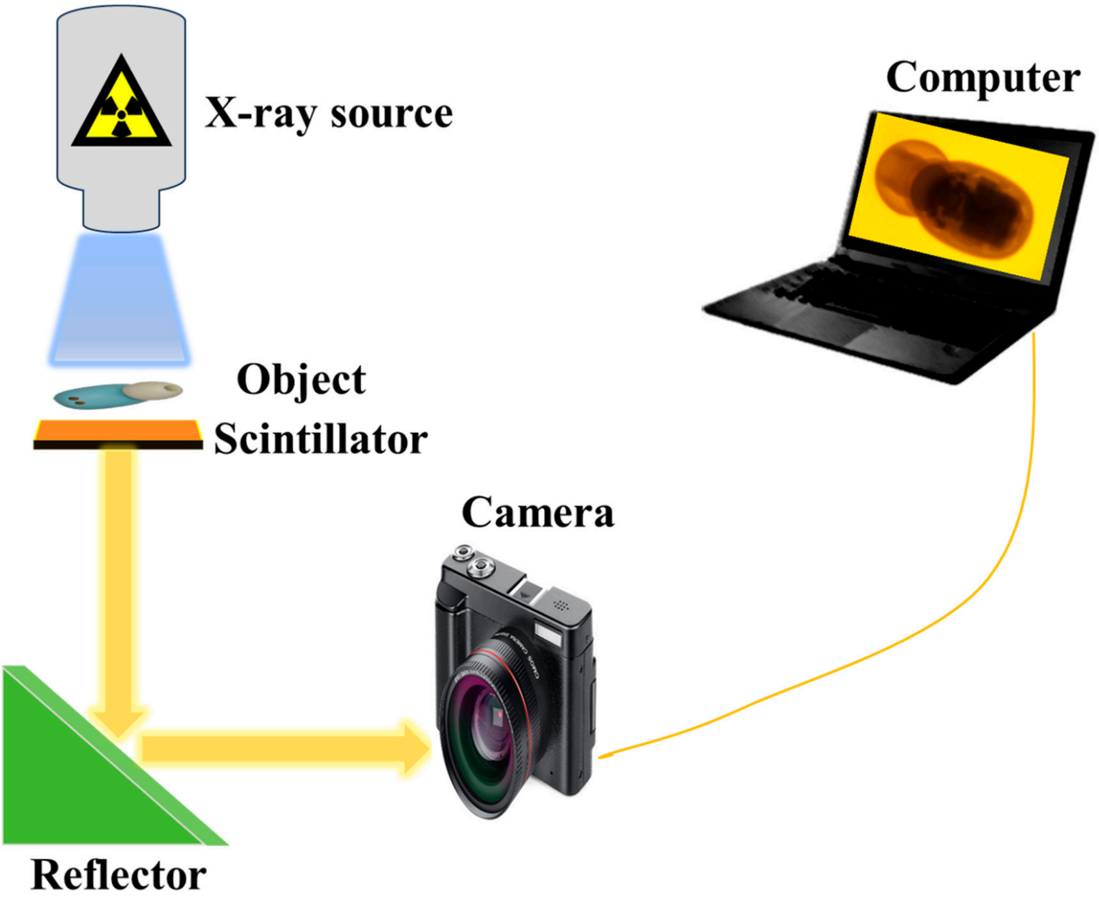

Figure S18. Schematic diagram of our self-built X-ray imaging system.

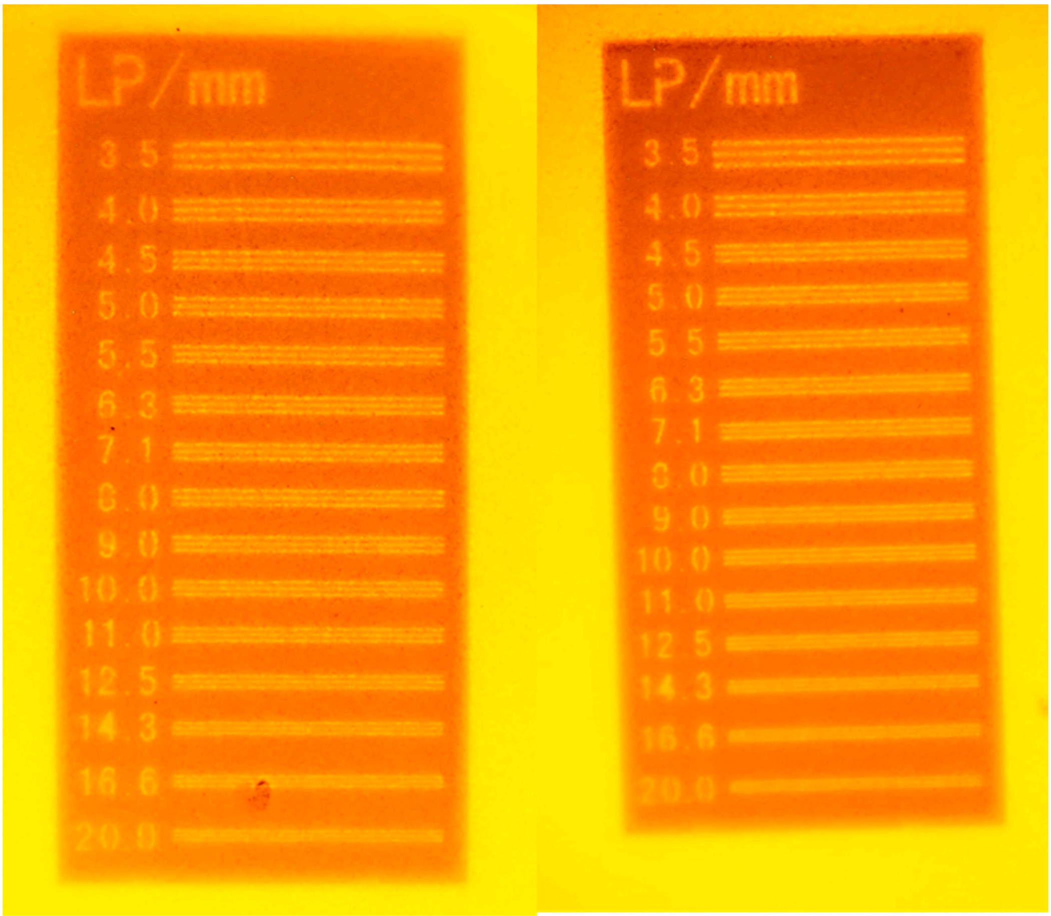

Figure S19. X-ray images of a Pb-based resolution scale, captured using two batches of $(DPPM)_2Cu_4I_4$@TPU scintillation films.

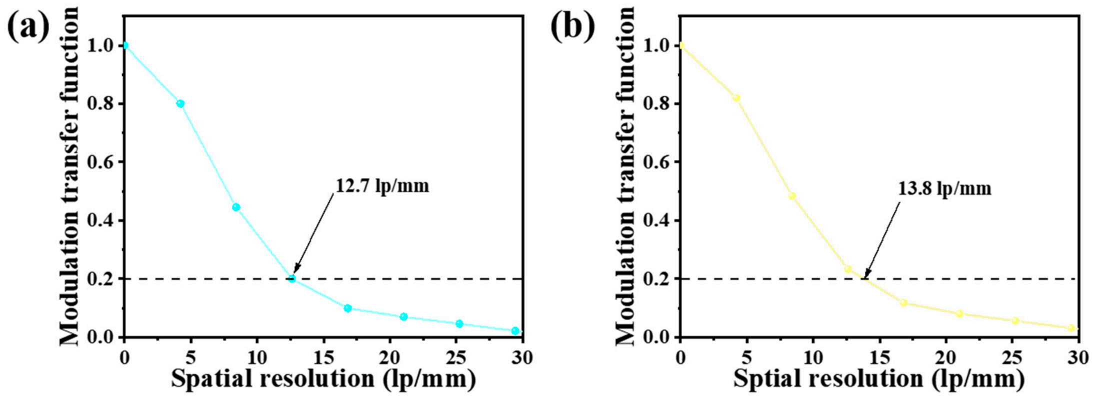

Figure S20. Modulation transfer functions of two batches of $(DPPM)_2Cu_4I_4$@TPU scintillation films: (a) sample 1, (b) sample 2.

Scintillators	PL peak (nm)	Preparation method	Detection limit (nGy/s)	Light yields (photons/MeV)	Resolution (lp/mm)	References
$(DBA)_4Cu_4I_4$	584	wafer	~	12842	5	[1]
$(C_8H_{20}N)_2Cu_2Br_4$	468	wafer	52.1	91300	9.54	[2]
$ETPA_2Cu_2I_4$	490	Powder-mixing	524	19900	5.47	[3]
$(PZ)_2Cu_2I_6.H_2O$	505,630	Powder-mixing	61	26700	16	[4]
$(MPP)_2SbCl_5$	570	Powder-mixing	532	28000	18.4	[5]
$CsPbCl_3:Yb^{3+}$	421,983	Powder-mixing	~	36740	13.6	[6]
$(BTPP)_2MnBr_4$	515	Powder-mixing	89.9	53000	10.1	[7]
$CsPbBr_3$	526	In situ	326	~	13.9	[8]
$(C_{24}H_{20}P)_2MnBr_4$	520	In situ	608	~	14.5	[9]
$(C_{19}H_{18}P)_2CuI_3$	492	In situ	194.3	26800	11	[10]
**$(DPPM)_2Cu_4I_4$**	**634**	**In situ**	**1566**	**17064**	**14**	**This work**

Table S1. Performance and materials characteristics of scintillators.

~ Not reported.

References

1. Hu, Q.; Zhang, C.; Wu, X.; Liang, G.; Wang, L.; Niu, X.; Wang, Z.; Si, W. D.; Han, Y.; Huang, R.; Xiao, J.; Sun, D., Highly effective hybrid copper(I) iodide cluster emitter with negative thermal quenched phosphorescence for X-ray imaging. *Angew. Chem., Int. Ed.*, **2023**, 135, e202217784.
2. Su, B.; Jin, J.; Han, K.; Xia, Z., Ceramic wafer scintillation screen by utilizing near-unity blue-emitting lead-free metal halide $(C_8H_{20}N)_2Cu_2Br_4$. *Adv. Funct. Mater.*, **2022**, 33, 2210735.
3. Zhan, Y.; Cai, P.; Pu, X.; Ai, Q.; Si, J.; Yao, X.; Bai, G.; Liu, Z. Exceptional optical performance of the zero-dimensional hybrid cuprous halide $ETPA_2Cu_2I_4$ as an X-ray scintillator. *Inorg. Chem. Front.* **2024**, *11*, 579–588.
4. Zhang, P.; Guan, H.; Li, C.; Du, Y.; Wang, C.; Wang, L.; Zheng, K.; Xiao, J.; Yan, Z., Undoped monophase hybrid copper(I) halides with highly efficient excitation-dependent dual-color emission for multiple applications. *Laser Photonics Rev.*, **2025**, 19, 2500171.
5. Wang, Y.; Cao, S.; Lai, J.; An, K.; Wu, D.; Chen, J.; Mu, H.; Wang, Z.; Guo, L.; He, P.; et al. Environment-friendly and highly efficiency organic antimony halide scintillator for light emitting diode and X-ray imaging. *J. Alloys Compd.* **2025**, *1024*, 180205.
6. Ran, P.; Yao, Q.; Hui, J.; Su, Y.; Yang, L.; Kuang, C.; Liu, X.; Yang, Y., Multi-energy X-ray linear-array detector enabled by the side-illuminated metal halide scintillator. *Laser Photonics Rev.*, **2023**, 18, 2300587.
7. Li, W.; Li, Y.; Wang, Y.; Zhou, Z.; Wang, C.; Sun, Y.; Sheng, J.; Xiao, J.; Wang, Q.; Kurosawa, S.; et al. Highly efficient and flexible scintillation screen based on organic Mn(II) halide hybrids toward planar and nonplanar X-ray imaging. *Laser Photonics Rev.* **2024**, *18*, 2300860.
8. Li, R.; Zhu, W.; Wang, H.; Jiao, Y.; Gao, Y.; Gao, R.; Wang, R.; Chao, H.; Yu, A.; Liu, X. Ultrastable and flexible glass−ceramic scintillation films with reduced light scattering for efficient X−ray imaging. *npj Flex. Electron.* **2024**, *8*, 31.
9. Xia, K.; Ran, P.; Wang, W.; Yu, J.; Xu, G.; Wang, K.; Pi, X.; He, Q.; Yang, Y.; Pan, J. In situ preparation of high-quality flexible manganese-halide scintillator films for X-ray imaging. *Adv. Opt. Mater.* **2022**, *10*, 2201028.
10. Cai, X.; Miao, X.; Bilal, M.; Wu, C.; Li, R.; Uddin, A.; Li, J.; Pan, J., In situ preparation of high-performance flexible copper halide scintillation films for X-ray imaging. *J. Mater. Chem. C*, **2026**, 14, 1624-1630.
